# The role of mouse tails in response to external and self-generated balance perturbations on the roll plane

**DOI:** 10.1242/jeb.247552

**Published:** 2024-11-06

**Authors:** Salvatore A. Lacava, Necmettin Isilak, Marylka Y. Uusisaari

**Affiliations:** Neuronal Rhythms in Movement Unit, Okinawa Institute of Science and Technology (OIST), Okinawa 904-0412, Japan

**Keywords:** Animal locomotion, Balance, Biomechanics, Vertebrate tail

## Abstract

Chordate tails exhibit considerable morphological and functional diversity, with variations in length, diameter and texture adapted to various ecological roles. While some animals, including humans, have lost or reduced their tails, many vertebrates retain and use their tails for activities such as balancing, climbing and escaping predators. This study investigates how laboratory mice (*Mus musculus*) use their tails to maintain balance when dealing with external and self-generated perturbations. Mice crossed platforms of different widths, while responding to roll-plane tilts. Our findings show that mice swing their tails to counteract external roll perturbations, generating angular momentum to stabilize themselves. Mice were also found to use active (dynamic stabilizer) and passive (counterweight) tail movement strategies when locomoting on narrow platforms. The results suggest that the tail is a core component of mouse locomotion, especially in challenging balancing conditions.

## Teaser

**Summary:** Mice use tail swings to counteract roll perturbations and maintain stability, highlighting the tail's crucial role in locomotion on challenging balancing conditions.

## INTRODUCTION

Tails represent a distinctive feature among chordates (Schwaner et al., 2021a). Despite their shared evolutionary and genetic developmental origins (Sallan, 2016; Young et al., 2019; Cumplido et al., 2024), tails show remarkable variety in their physical characteristics. They can vary significantly in length, diameter and texture, ranging from short and slender to long and robust (Hickman, 1979). Throughout evolution, mammalian tails have diversified for various purposes, such as crawling, swimming, hopping and climbing. Humans and numerous other animals have lost their tails (Xia et al., 2024); however, a significant portion of vertebrates have retained, lengthened, enriched or repurposed their tails. Many animals such as felines (Walker et al., 1998; Patel et al., 2016), primates (Larson and Stern, 2006; Massaro et al., 2016) and rodents (Sheard et al., 2024; Kingsley et al., 2024) have long tails that help in maintaining balance and flexibility at high speeds. An interesting example of tail use in balancing can be observed in the kangaroo rats, which swing their tails over their backs during high leaps. This dynamic tail use is thought to counteract sudden changes in the angular momentum of the body, preventing the animal from flipping during the aerial phase (Bartholomew and Caswell, 1951). Kangaroo rats also use their tails to maintain appropriate posture and balance while hopping (Bartholomew and Caswell, 1951) and when escaping from predators (Schwaner et al., 2021b).

A recent review from Mincer and Russo (2020) explored the factors influencing the evolution of mammalian tail morphology. Their phylogenetic comparative method supports the idea that arboreal habitats may favor longer tails. In particular, species residing in arboreal environments have longer tails and tail loss is more prevalent in non-arboreal species (Mincer and Russo, 2020).

During locomotion on a narrow substrate such as a tree branch, animals need to maintain their center of mass (CoM) above a narrow base of support where even small perturbations in the roll plane (perpendicular to the main axis of the animal body) may lead to loss of balance. A tail can be used to improve stability during locomotion in such conditions in two main ways: passively (e.g. by lowering the CoM) or actively (e.g. by generating compensatory angular momentum to counteract undesired body rotations; Larson and Stern, 2006). Despite being light in weight, a long tail could potentially generate substantial angular momentum if moved at high speed as the moment of inertia increases with the square of length. This insight is in line with the observation that arboreal species (for whom maintaining balance on thin branches is critical for survival) have longer tails compared with terrestrial species.

In this work we examined how laboratory mice (*Mus musculus*) use their tails to respond to balance perturbations in the roll plane. Mice have a relative long tail (7 cm on average) and a natural ability to walk across narrow beams even when exposed to the task for the first time (our personal observations). Firstly, we addressed this question in the context of an external perturbation (tilt on the roll plane), where we predicted that if the tail is used to counteract such perturbations then it will rotate in the roll plane and the direction of the response will depend on the direction of the tilt. Secondly, we investigated whether mice could use their tails in response to self-generated perturbations. We hypothesized that during locomotion on a narrow substrate mice could maintain stability by either lowering their tail position and keeping it static so as to lower the whole-body CoM (passive strategy) or by extending the tail to the side (thereby increasing the moment of inertia in the roll plane) and producing large tail rotations to mitigate undesired body rotations. Finally, we examine whether the tail movements adapt to changes in the width of the locomotory substrate, with the expectation that on narrower and more challenging platforms the role of the tail in locomotion will become more prominent in comparison to its role on a flat surface (Machado et al., 2015).

## MATERIALS AND METHODS

### Animals

All procedures were reviewed and performed in accordance with the OIST (Okinawa Institute of Science and Technology) Graduate University's Animal Care and Use Committee (protocol 2020-292-2). C57BL/6 mice were purchased from Clea (Japan) and were acclimatised to the OIST facility for at least 1 week before handling. They were housed in institutional standard cages (5 animals per cage) on a reversed 12 h light:12 h dark cycle with *ad libitum* access to water and food. At the commencement of the handling period, the mice were 8 weeks old.

### Anatomical measurements

Measurements of body and tail mass and length were obtained from six mice carcasses. These mice were of the same sex, strain and age (10–12 weeks) as those used in the experimental recording. The weight of these mice and the experimental group was compared to ensure the anatomical measurements extracted from the carcasses can be generalized to the experimental group, and no significant difference is shown (unpaired *t*-test; *t*_19_=0.27, *P*=0.7922). The center of mass (CoM) was determined from these carcasses utilizing a reaction board, adapting the method described in Hay and Reid (1988). After estimation of the CoM, the tail was dissected at its base from the body and the weight and length of the tail and the body were measured.

### Animal training

After a 2-week handling and habituation period, mice were trained to cross the platform using the 5 mm (days 1, 3, 5 of training) and 8 mm (days 2 and 4) ridges. The mice were gently encouraged to traverse the ridge, and only when necessary, were provided additional incentive for traversing by placing a familiar object from their home cage in front of them. No food or other rewards were used, and the animals were not incentivised artificially (e.g. with food deprivation or stressors). Each training session lasted until the mouse could cross the platform without stops 10 times in a row. Each training session lasted for approximately 2 h. Each mouse underwent such training for five consecutive days. All 15 mice used in this study successfully acquired the task and traversed the ridge at the end of the 5th training day.

### The ridge task

**Fig. 1.**
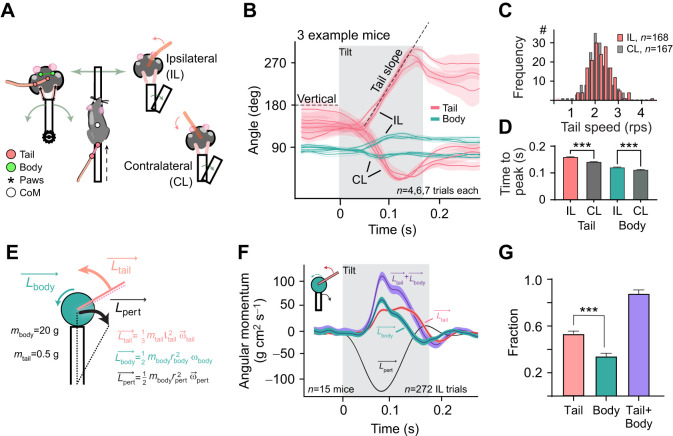
**Roll-plane tilting evokes counteracting tail responses.** (A) Schematic of the ridge task. Left: tracked body parts from rear and top views. Right: ‘ipsilateral’ (IL) and ‘contralateral’ (CL) trials are defined by whether the tilt matches the tail position direction. (B) Tail and hip angle trajectories (pink and green) from three example mice. Gray area indicates ridge movement. (C) Comparison of mean tail acceleration in IL versus CL trials. (D) Comparison of tail and hip movement duration in IL versus CL trials. (E) The biomechanical model estimating angular momentum experienced by the mouse and compensation from body and tail rotation based on tracked movements (see Eqns 1–3 in main text for further details). (F) Instantaneous momentum estimated by the model. Traces show means±s.e.m. for *N*=15 mice for tail, body and their sum, along with the estimated perturbation-generated rotational momentum. Downwards direction corresponds to the tilt direction. (G) Total angular momentum generated by tail, body, and their sum as a proportion of perturbation-generated momentum. Data shown as means±s.e.m., compared using *t*-test (B) or one-way ANOVA (D) with Bonferroni's post-test (***P<0.001).

A diagram illustrating the ridge task is shown in Fig. 1A. The ridge set-up consists of a thin acrylic ridge (4–10 mm wide, 50 cm long) attached on one end to a motor (S3003 Servo Motor, Futaba, Japan) controlled by a Bonsai script (https://bonsai-rx.org/) that can tilt the ridge left and right direction with varying amplitudes. A small platform was placed at both ends of the ridge for the mouse to comfortably reside before and after the trial. Mouse movement was recorded at 300 FPS with two high-speed cameras (Blackfly S USB3, Teledyne FLIR, Wilsonville, OR, USA), one placed 50 cm above the ridge and another at the rear end of the ridge. Video recordings were saved in mpg format.

### Experimental trial structure

During the experiment, mice were subjected to trials with a tilt perturbation randomly intermingled with non-perturbation trials, and the onset of the tilt was randomly timed during a trial to prevent anticipatory behavior. After the tilt, the ridge remained in that position through the remaining trial. The baseline perturbation trials involved a 20 deg (190 ms) tilt of the ridge in either the left or right direction.

Four different ridge widths (4, 5, 8, 10 mm) were used to provide mice with different challenging tasks, as well as a 45 mm non-tilting ridge that allowed comparison of ridge-traversing locomotion to ‘flat surface locomotion’. In additional trials the perturbation angle was either decreased or increased [to 10 or 30 deg (for 150 or 230 ms), respectively] without changing the angular velocity of the tilt. The trials with different ridge widths or tilt amplitudes were randomized according to a Latin square design. The entire experimental series was completed in 15 experimental days.

### Tracking tail and body movements

Videos were recorded with the top and rear cameras as described above. The nose, tail, body and hind paw trajectories were extracted using DeepLabCut (v. 2.1.7; https://github.com/DeepLabCut/DeepLabCut; Mathis et al., 2018). A total of 200 image frames (10 videos selected from perturbation/non-perturbation trials, as well as different widths, 20 frames per video) were used to label and train models from both camera views; 90% of the labeled frames were used for training, and the remaining 10% for testing. We used a ResNet-50-based neural network for 1,000,000 training iterations, where the cross-entropy loss plateaued to 0.001. We then used a *P*-value cut-off of 0.9 to condition the *x*,*y* coordinates for future analysis. This network was then used to analyze videos.

In addition to the hind body kinematics, position of the body centroid was estimated using the top camera view as the average position of the mouse body silhouette using a custom-made script in Bonsai. Silhouette was extracted by first applying a filter to binarize the image [to separate the region of interest (ROI) from the background], and then extract the ROI centroid, as described in Lopes et al. (2015). The point in time when the ROI centroid became visible under the top camera was used to extract the time-aligned traces captured by both cameras. Out of this trace, the 500 points (centered in time) of the time series were used to compute angles displacement during a trial. Tail and body angle time-series were either extracted for the trial, or for a step cycle. A step cycle was identified based on the peak of the *x* projection time-series of the contralateral (with respect to the position of the tail) hind paw marker. This time point was used to separate the trials based on step events and to project the time-series of body and tail angles centered in time around this event.

### Kinematics analysis

Custom Python scripts (Python 3.7.4; https://www.python.org run on Windows 10) were used to compute angles and instantaneous velocities for all DeepLabCut-extracted markers, as well as the speed of the Bonsai-extracted centroid trajectories. All time series were applied a smooth filter (Hanning smoothing) of 10 frames (33 ms) before further processing.

The following parameters were computed from the extracted trajectories. (1) Roll-plane tail angle: the angle of the initial segment of the tail with respect to vertical as seen from the posterior camera, where 180 deg corresponds to tail pointing up (Fig. S1A). (2) Yaw-plane tail angle: the angle of the initial segment of the tail with respect to the ridge, as seen from the top camera; 0 deg corresponds to tail pointing straight back (Fig. S1A). (3) Hip angle: alignment of a line connecting the two hip markers with respect to vertical, as seen from the posterior camera; 90 deg corresponds to horizontal alignment (Fig. S1A). Instantanenous angular velocity is the frame by frame gradient of the tail angle. (4) Back angle is the angle between the tail base, the centroid and the line parallel to the ridge passing through the tail base. It is used to estimate the mouse back posture while crossing the ridge; 0 deg corresponds to the hind body being aligned straight back along the ridge (Fig. S1A). (5) Front angle is the angle between the centroid, the nose, and the line passing through the nose and parallel to the ridge; 0 deg corresponds to the head being aligned straight ahead along the ridge (Fig. S1A). (6) Tail-on-body angle is obtained by subtracting the back angle from tail angle (determined by tail second marker, tail base and the line parallel to the ridge passing through the tail base) (Fig. S1A); 0 deg corresponds to the tail being aligned straight back along the ridge.

### Ridge traversing performance assessment

Performance was assessed using 5 metrics: slip count, traversing speed, duration CoM (center of mass) was outside the base of support (BoS), CoM lateral movement and CoM centrality. Paw slips, defined as the slip of at least one paw from the platform, were manually counted from slow-motion videos acquired at 300 Hz. Traversing speed was calculated as the forward distance covered by the centroid (measured from the top camera) divided by the time it took to cover that distance. CoM outside BoS duration for one trial is defined by the time that the centroid was outside the edges of the ridge. CoM lateral movement during a trial was calculated as the mean absolute distance of the centroid from the midline of the ridge. Finally, CoM centrality is the lateral distance of the centroid normalized by the width of the ridge (where 1 represents the case where the centroid is on the midline and 0 on the edge).

### Biomechanical model

A simple biomechanical model was constructed to estimate the angular momentum contributions of the tail, body, and perturbation (see Fig. 1E). The tail was approximated as a uniform rod of length 7 cm and mass 0.5 g, while the body was modeled as a cylinder with a diameter of 2 cm and mass 20 g (weights and size based on measurements of 6 male mouse carcasses as described above). These simplifications allowed for straightforward computation of the angular momentum, making the model analytically tractable.

The angular momentum for the tail, body, and perturbation were computed using the following formulae:
(1)

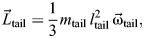

(2)

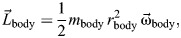

(3)

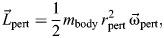
where *m* is the mass (of the body or the tail), *l* is the length of the tail, *r* is the radius (of the body or the platform), and ω is the angular velocity (of the tail, body, or platform). The tail was modeled as a uniform rod rotating about its perpendicular axis (Eqn 1), assuming that the tail's mass is evenly distributed along its length. For the body (Eqn 2), the cylinder approximation assumes the mass is distributed symmetrically about the central axis. The perturbation (Eqn 3) is modeled similarly to the body, as it involves rotation about the central axis. It estimates the momentum experienced by an object (in our case the mouse) placed at the edge of a tilting platform.

### Cross-correlation analysis of tail and hip momenta

The cross-correlation was calculated between body and tail momentum time bins for trials with a certain width. ‘Hotspots’ and ‘coldspots’ were defined by collecting cells with values in positive and negative 5th percentile for the total distribution of correlation and highlighting region with the highest number of contigous cells.

### Statistical analysis

All data are presented as means±s.e.m. Data were analyzed using one-way analysis of variance (ANOVA), as appropriate. Bonferroni test was used for *post hoc* analyses of significant ANOVAs to correct for multiple comparisons. Differences were considered significant at the level of *P*<0.05. Statistical analysis was performed with Prism 9.0 (GraphPad, San Diego, CA).

## RESULTS

### Tail rotation generates significant momentum

While traversing the ridge, mice tended to hold their tails on one side (i.e. not parallel to their body axis, Movie 1). We first examined ridge trials where the tilt occurred in the direction of the tail position [‘ipsilateral (IL) tilt’; Fig. 1B]. The tilt elicited not only a moderate back-and-forth motion of the body (Fig. 1B, green) but also a rapid tail swing to the opposite side with remarkably invariant kinematics (Fig. 1C,D, red). Tilts that were directed to the opposite side of the tail [‘contralateral (CL) tilt’; Fig. 1B] also elicited tail responses with mirrored kinematics and nearly identical speed profiles (IL, 2.20±0.037 rotations per second (rps); CL, 2.12±0.038 rps; *P*=0.15) even though the range of movement was limited by the tail making contact with the ridge (Fig. 1B–D). Tail angles are shown based on the initial tail segment (Fig. 1A), as the tail swing motion starts from the tail base (Fig. S1A,B), and the tail is kept straight at the tilt onset, and the difference in tail base versus tail tip angles approaches 90 deg only at the end of the tail response (Fig. S1A).

For quantitative analysis of the response, we used a biomechanical model to calculate angular momentum for tail, body and external perturbation are illustrated in Fig. 1E. As the contralateral tail movements were limited due to contact with the ridge (evidenced in the lower movement range in Fig. 1E) and as the resulting compensatory momentum greatly depends on the position of the tail at the onset of the ridge tilt, we only consider ipsilateral trials for the rest of this work.

Despite weighing only a fraction of the body mass (2.56±0.12% of the body without a tail), the observed movement of mouse tail results in compensatory momentum with peak magnitude comparable to that generated by rest of the body (Fig. 1F). The total tail-generated momentum was larger than that of the body during the tilt (Fig. 1G). This was due to the high tail angular velocity as well as the longer duration of tail versus body momentum (time to peak position for tail and body during IL tilts 0.16±0.0014 and 0.12±0.0016 s, unpaired two-sample *t*-test, *P*<0.001). Ultimately, the sum of compensatory momentum by body and tail amounted to over 80% of the total estimated rotational momentum experienced by the body in response to the ridge tilt (Fig. 1G, blue).

### Tail swings in response to different tilt conditions

**Fig. 2.**
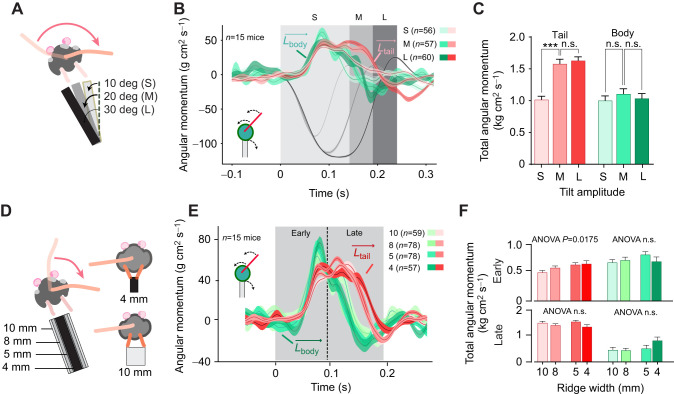
**Effect of tilt duration and ridge width on tail response to external perturbations.** (A) Time course of tail (red) and body-generated (green) angular momentum opposing tilt-induced momentum (gray). Shading denotes tilt durations and time windows for total momentum calculations in B and C. (B) Total angular momentum generated by tail and body during tilts. (C) Total momentum for tail, body and their sum as a fraction of tilt-induced momentum. (D) Schematic showing hind paw alignment on narrow and wide ridges. (E) Time course of tail and body angular momentum in response to medium-duration tilts on ridges of different widths. Shaded area indicates tilt duration; dashed line divides early and late response phases used in F. (F) Total momentum of tail response increases on narrower ridges during early (top) but not late (bottom) response phase. Data are means±s.e.m., with comparisons using one-way ANOVA followed by Bonferroni's post-test (***P<0.001).

The diagram in Fig. 2A illustrates the three tilt angles used, 10, 20 and 30 deg [which from now on are referred to as small (S), medium (M) and large (L) condition]. Firstly, we measured the effects of the tilts on the forward speed of the mouse (see Fig. S2A). The performance of mice experiencing either 10 or 20 deg tilts were indistinguishable, but the largest (30 deg) tilt posed a greater challenge and lead to complete stopping (defined as forward speed less than 1 mm s^−1^, previously defined as immobility threshold in Yang et al., 2020) and a significant reduction in the distance the mice traversed in the 0.5 s time window after the tilt (Fig. S2A3; Table 1). The time courses of tail and body-originating compensatory momentum show that the tail swing duration was shortest in the S condition, but both tail and body maintain similar trajectories across conditions (Fig. 2B). The plot in Fig. 2C shows no difference in the generated momentum from the body in response to different tilts, and a smaller tail momentum during short tilts. We also computed the relative angular momentum (total momentum normalized by the external perturbation, Fig. S2B), which shows a gradual decrease in body and tail relative contribution to tilt compensation, with L condition being way below 1 (0.61±0.020, Table 2, Fig. S2B).

**Table 1.**
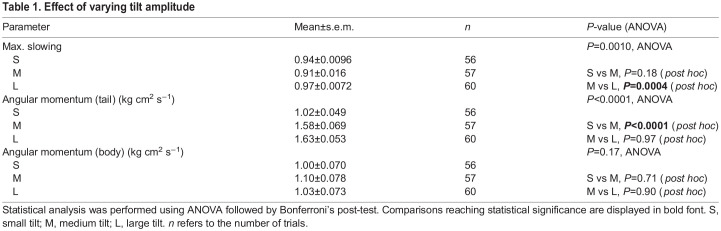
Effect of varying tilt amplitude

**Table 2.**
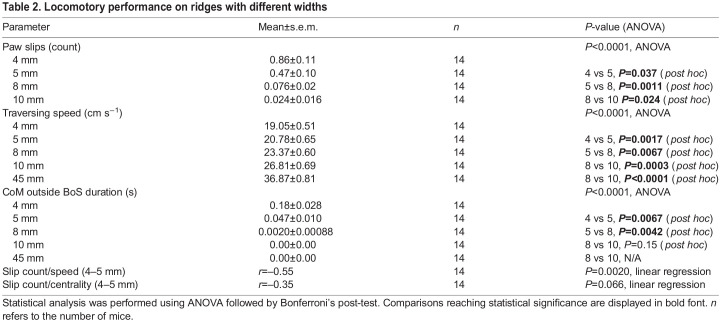
Locomotory performance on ridges with different widths

By using ridges of different widths (4, 5, 8 and 10 mm) we observed mice crossing the ridge with subtle changes in posture (Fig. 2D; Movie 2). We found a slight trend for higher tail-generated compensatory momentum on narrow ridges (ANOVA, *P*<0.5; Fig. 2E,F; Table 2) during the early phase of the response, whereas no effects were seen in momentum generated by body rotation. No differences were found either in body or tail-originating momentum in the late phase of the response (Fig. 2F, bottom).

### Balance performance and body posture in narrower ridges

**Fig. 3.**
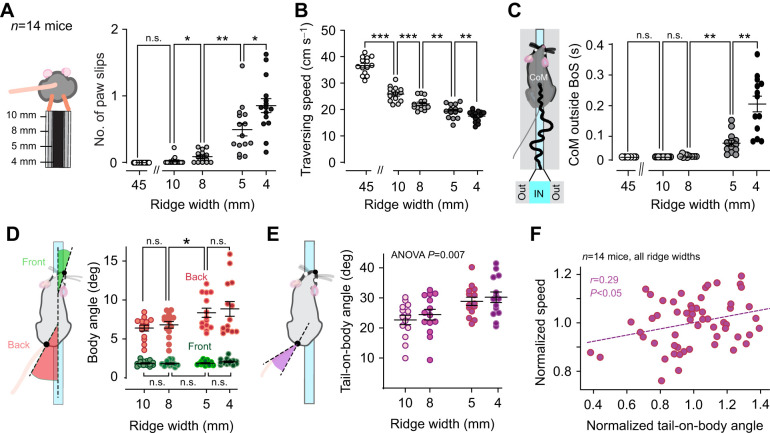
**Effect of ridge width on body posture and task performance.** Classical quantification of ridge traverse performance by number of paw slips (A) and traversing speed (B). (C) Duration for which the center of mass (CoM) was entirely within the base of support (BoS), as determined by the ridge edges (schematic on the left). (D) Schematic (left) and summary of mean alignment angles of front and hind-body with respect to the ridge. (E) Schematic (left) and summary of mean tail angles with respect to the hind-body angle. (F) Larger tail-on-body angles were correlated with better performance in terms of traversing speed. All data are presented as means±s.e.m. and were subjected to statistical analysis using one-way ANOVA followed by Bonferroni's post-test (*P<0.05, **P<0.01, ***P<0.001).

Locomotory performance of mice on ridges with different widths (without tilt) is shown in Fig. 3A–C. We found an increase in paw slips (measured by manual counting) for narrower widths (Fig. 3A; Movie 3; Table 2). Next, we showed that the traversing speed gradually decreases in narrower ridges, with the biggest difference being shown between the flat surface and the 10 mm ridge condition (Fig. 3B). As shown in Fig. 3C, the animals maintained their CoMs within the edges of the platform (acting as a base of support, BoS) throughout nearly all trials except on the most narrow (4 mm) ridge (see Table 2 for all values). By measuring the normalized and absolute centroid distance from the center of the ridge (CoM centrality and lateral movement, respectively), we revealed more gradual changes across the different widths, with narrower ridges leading to a decrease in centrality (Fig. S3A). Furthermore, we compared these metrics of performance and showed that mice with more paw slips tend to cross the ridge at lower speed and centrality (Fig. S3B).

On all ridges, mice maintained the alignment of their head and front body with the ridge (Fig. 3D, green). However, on 5 and 4 mm ridges the caudal body posture shifted to the side (Fig. 3D, red; see also Movie 3). The angles of either front or hind-body did not show any significant correlation with speed or CoM centrality measurements (Fig. S3C). In addition to the angled hind-body posture, mice also held their tails closer to perpendicular with respect to the ridge when traversing 4–5 mm ridges (Fig. 3E). Mice traversed the ridges faster during trials in which they held their tails at high angles (normalized to ridge-group means; Fig. 3F), but no relation was found between the tail angle and CoM centrality (Fig. S3D; see Table 3 for all values).

**Table 3.**
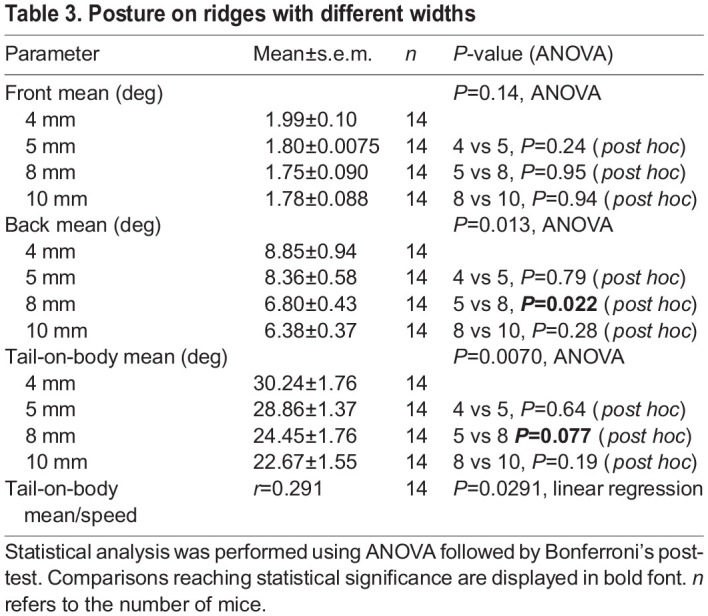
Posture on ridges with different widths

### Tail kinematics in narrow-substrate locomotion

**Fig. 4.**
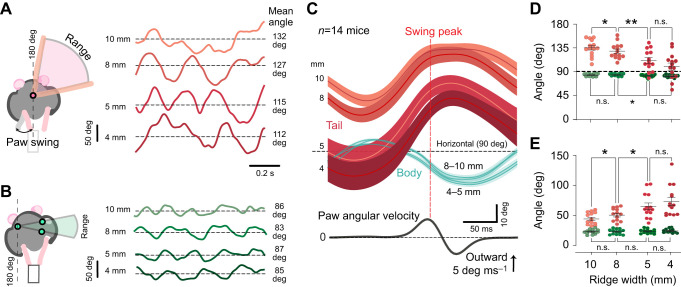
**Tail and hip movements during unperturbed ridge traverse.** Rear-view angle trajectories of tail (A, red) and body (B, green) angles of an example mouse traversing ridges of different widths. One second of the trials is shown starting from the beginning of ridge traverse. Dashed lines indicate the mean position for the tail in a given trial. Schematics on the left depict the measurements. (C) Tail and body movements temporally aligned on the contralateral paw swing. Mean±s.e.m. angle trajectories of tails (red) and body (green) of 14 mice. Bottom panel shows average of contralateral paw angular velocity, aligned on the outward peak. (D) Mean position of the body and tail. Data are shown as averages of single animals over all trials of a given width. (E) Range of tail and body motion through step cycles. (*P<0.05, **P<0.01, n.s., *P>*0.05).

Representative trials for the roll-plane motion of mouse tails and body while mice cross different ridges are shown in Fig. 4A,B, respectively. Periodic oscillatory movements in both tail and body were also apparent in single traces. These oscillations are shown more clearly in Fig. 4C, where we aligned in time the traces to the contralateral hindlimb step cycle. While body movement remains remarkably similar in terms of position and rotation range (Fig. 4D,E, respectively), the tail moves closer to the horizontal axis in more challenging conditions (Fig. 4C,D). The tail rotational range also increased almost gradually when going from wider to narrower ridges (Fig. 4C,E).

**Fig. 5.**
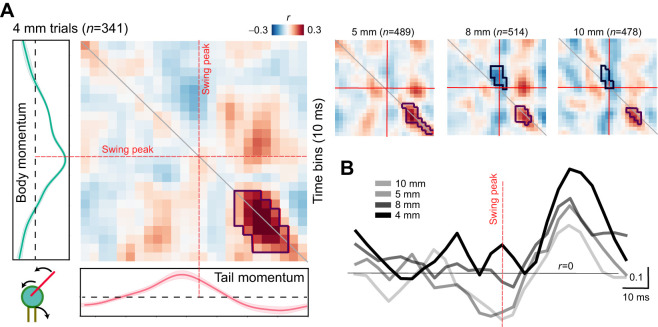
**Tail and hip cross-correlation during step cycle.** (A) Cross-correlation between the tail and body momentum through the step cycles centered on swing peak. Left, cross-correlogram for the 4 mm ridge condition. Cross-correlation values are displayed in a normalized intensity range between 0.3 (red) and −0.3 (blue). Cross-correlograms for 5 mm, 8 mm and 10 mm ridges are shown as smaller panels to the right. ‘Hotspots’ and ‘coldspots’ (see Materials and Methods) are depicted with dark contours. Dashed red lines indicate swing peak times. (B) Mean correlation values along the diagonal for each ridge width. Darker lines correspond to trials with narrower ridges. Red dashed line indicates time of swing peak.

When examining the cross-correlation between hip and tail oscillations on the 4 mm ridge (Fig. 5A, left; high correlation region indicated with red outline) we found that their movements are most strongly coupled shortly after the contralateral paw's stance onset. This coupling is most pronounced when the mouse is traversing the 4 mm ridge, and diminishes on easier ridges (Fig. 5A, right panels). In contrast, the coupling between the tail and body showed increasingly negative correlation in broader ridges (Fig. 5B, right panels; blue outline), that is centered around the peak swing phase (cross-correlation values shown along the matrix diagonal in Fig. 5B).

## DISCUSSION

In this study we show that mice rotate their tails in response to external and self-generated roll-plane perturbations. Examining the kinematics of the tail using a tractable biomechanical model, we show that such tail rotations can generate significant amount of angular momentum that complements other postural adjustments (such as positioning the tail low as a counterweight) aimed at maintaining balance.

Despite the fact that our mice had no previous experience of such tasks, all individuals produced these corrective tail swings from the first trial, attesting to the relevance of this balance response in their behavioral repertoire.

### Tail function in response to an external perturbation

We examined whether mice responding to an external perturbation use their tails simply as a counterweight (‘passive use’), or if they take advantage of the tail length and its range of motion to generate compensatory angular momentum (‘active use’). If the tail is only used as a counterweight, we predicted that in contralateral trials (where the tail is already placed opposite to the tilt) we should not observe any rotational movement. However, we found that tail rotations were generated in both conditions, always towards the direction countering the tilt (Fig. 1A). Importantly, the swing kinematics were remarkably similar in both directions, despite being limited by contact with the ridge in contralateral trials (Fig. 1B). This indicates that the tail rotation in itself is a core component of the balancing strategy. To quantify the effect of the rotation on mitigating the external tilt we used a tractable biomechanical model to estimate the magnitude of angular momentum generated by the tail (Fig. 1C). To our surprise, tail rotational momentum was estimated to account for over 50% of attenuation of the perturbation, significantly more than that estimated to be generated by the concomitant body rotation (Fig. 1D).

Next, we introduced changes in the tilt angle to test the adaptability of the tail response. While the angular momentum generated by the body rotation did not increase with larger tilts, the tail momentum was significantly higher when tilts were increased from to 10 to 20 deg (Fig. 2B,C). This suggests that the tail can offer a selective advantage in responding to external tilts of different intensities, and that this response cannot be explained simply as a passive reaction to body rotations. Notably, in the 30 deg tilt trials, the tail angular momentum may have been limited by the contact with the ridge, which could explain the decreased locomotory performance of the mouse (Fig. S1).

To investigate whether the tail swing and body rotation responses are modulated when the perturbations are presented in different proprioceptive contexts, we repeated the experiments using ridges of different widths (4, 5, 8 and 10 mm) where mice crossed the platform with subtly but significantly different postures (Fig. 2D–F). Focusing on the early phase of the compensatory responses that is most likely to be affected by the different body configuration, we found only a slight trend towards higher tail momentum on the narrowest ridges (Fig. 2F), while no effects were observed on the body momentum. These observations suggest that the tail-swing offers a balancing strategy that is most useful under challenging conditions where body rotation or paw placement cannot provide sufficient compensation. However, the kinematic characteristics of the tail swing are remarkably similar across changes in proprioceptive contexts, suggesting an all-or-none motor command that is activated under specific conditions.

### Active and passive tail use during self-generated movements

The next question addressed in the study was how mice use their tails to stabilize themselves during unperturbed locomotion in a challenging balancing environment. Particularly, we speculated that the tail could help mice improve balance by either lowering their CoM (passive strategy) or producing a momentum to mitigate undesired body rotations (active strategy). We found that mice tails produce oscillations phase-locked to their step cycle, and these oscillations become bigger in amplitude while mice crossed a narrower ridge (Fig. 4C), which is in line with an active role of the tail in mitigating body rotations. However, we also found that on the narrowest platforms (4 and 5 mm) mice lower their tails, so that at least during part of their oscillations they are placed at an angle below 90 deg (Fig. 4D), suggesting that the tail can be used passively to lower the CoM. Taken together these results highlight that while in all ridges mice produce active oscillations that counteract undesired body rotations in the roll plane, in the most extreme balancing conditions mice also could further stabilize their bodies by lowering their tails and CoM.

Finally, when examining the body and tail momentum together (Fig. 5), we found that they were most strongly coupled during the stance onset of the contralateral paw, and the magnitude of the coupling increased on narrower ridges. On the other hand, tail and body momentum are negatively correlated around the swing peak phase of the contralateral paw (i.e. when the mouse is in a precarious phase of the stepping cycle) on the wider platforms (8 and 10 mm). In particular, this last observation is in line with the tail function of an active stabilizer, where larger tail momentum is associated with lower body momentum during the swing-phase of the step-cycle.

Although our relatively simple recording and modeling methods (e.g. not accounting for ground forces and limb torques) limit the conclusions we can draw regarding tail movements in supporting locomotion, our findings strongly suggest that the mouse tail acts as a dynamic stabilizer under challenging conditions. Specifically, by engaging in context-dependent oscillatory movements, the tail appears to actively counteract body oscillations, reducing the risk of falling and enabling the mouse to maintain balance while advancing at high speeds (Shield et al., 2021).

### Revising metrics and targets for studying balance in rodents

Maintaining balance is a complex task where an animal has to engage continuous, contextually appropriate motor plans in an ever-changing environment. However, past work on balance assessment in laboratory mice is largely based on coarse measurements, such as the number of falls from a rotarod (Carter et al., 2001; Stanley et al., 2005). Such measures treat balance as a discrete variable, where the animal can only be in either of two states (balanced or fallen) without acknowledgement of the subtler features of balancing strategies.

In this work, where mice typically never fell off the ridge, we defined ‘balance performance’ as a metric examining position of the mouse center of mass with respect to the base of support. Comparing this measure with two conventional metrics of balance performance (paw slips and traversing speed), we find them in good general agreement with each other. However, noting that paw slip counts are cumbersome to generate (Wahl et al., 2023 preprint) as well as subject to confounding factors such as substrate surface properties, we highlight that the CoM trajectory provides a nuanced insight into an animal's balancing performance and offers a methodologically simple yet robust view of the animal's balance capabilities (Latash et al., 2020). We expect that combining CoM-based balance assessment with other metrics (such as traversing speed and number of stops; Modi et al., 2023) will prove to be an insightful approach to dissect balancing strategies in the context of specific motor aberrations.

Combining these approaches and assessing locomotory performance on the range of ridge widths (4, 5, 8 and 10 mm), we were surprised by the differences detected when the width differed by only 1 or 2 mm. Conventionally, studies on mouse balance have used a beam of a fixed width (Jamwal et al., 2017; Mann and Chesselet, 2015). Hence we call for caution to future studies employing only single-width beam to measure balance performance, as subtle features of balancing performance can be missed, especially if limited to relatively broad (1 cm) beams.

### Conclusion

Our study shows that the tail plays an important role in mouse locomotion, with distinctive strategies to modulate external and self-generated perturbations. The tail response to an abrupt external tilt is an all-or-none swinging motion engaged whenever a roll-plane perturbation is sensed, generating substantial amount of angular momentum. Similarly, the tail oscillates anti-phase with respect to self-generated body rotations during locomotion in narrow platforms, supporting the idea that mice could use their tails as an active stabilizer. In summary, we underscore the tail's vital function as a ‘fifth limb’ providing biomechanical support and enabling mice to adapt effectively to changes in the environment, and call for further attention to the mouse tail in future works focusing on balancing behavior.

## Supplementary Material

10.1242/jexbio.247552_sup1 Supplementary information
