# Supplementary material for: The role of mouse tails in response to external and self-generated balance perturbations on the roll plane
Source: J Exp Biol. 2024 Nov 6;227(21):jeb247552. doi: 10.1242/jeb.247552 (PMC11574348; doi:10.1242/jeb.247552)
Supplement: Supplementary information [file jexbio-227-247552-s1.pdf]

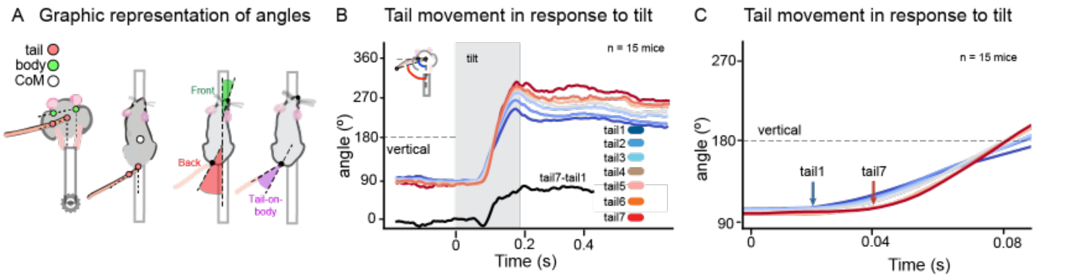

Fig. S1. Tail segments angles during roll tilt. A, Graphic representation of tail and body angle tracked from the posterior camera (left panel), as well as angles and centroid tracked from the top camera (right panels). B, Tail angles of diAerent tail segments (1 being the initial and 7 being the most distal) in response to tilt for ipsilateral trials (n = 15 mice). C, expanded plot on the time axis to highlight the delays in swinging motion initiation between the Ærst and last segment (indicated by blue and red arrows respectively). Gray area indicates ridge movement.

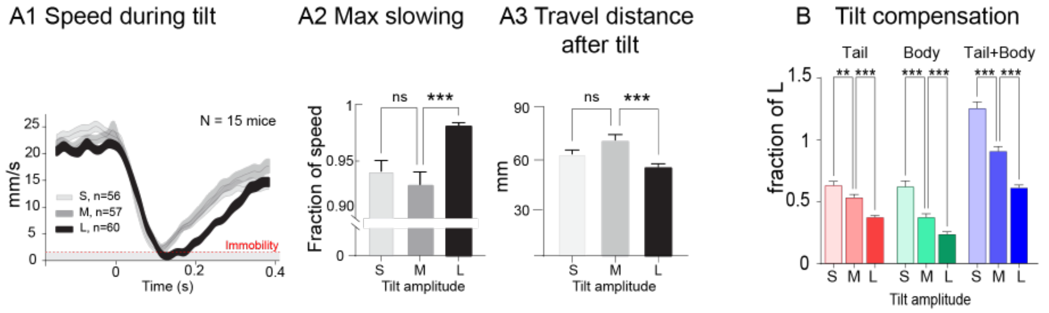

Fig. S2. Tilt duration and ridge width eAect on tail response to external perturbations. A1, decrease in forward velocity of the mice during perturbations. Dashed line indicates threshold value for immobility (1 mm/s). A2, eAect of larger tilt on task performance quantified as extent of slowing from pre-tilt-velocity. A3, distance travelled during the 0.5 sec following a tilt. B, total momentum for the tail, body, and their sum as a fraction of the total tilt-induced momentum. Data are presented as the mean ± SEM, and statistical comparisons were conducted using oneway ANOVA followed by Bonferroni's post-test (*p<0.05, **p<0.01, and ***p<0.001).

Journal of Experimental Biology • Supplementary information

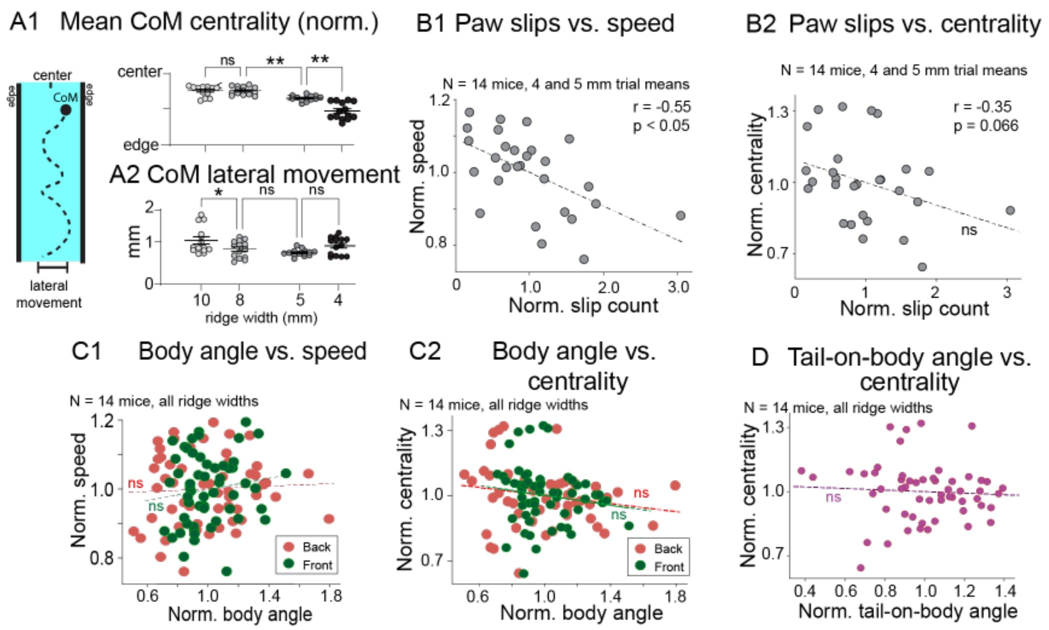

Fig. S3. Effect of ridge width on body posture and task performance. A1, left: CoM centrality ranges from 1 (center of the ridge) to 0 (edge or beyond). Right, top: CoM position is less central on narrower ridges. Bottom: lateral CoM movement amplitude is consistent across ridge widths. C, balancing performance metrics: C1, paw slips versus speed; C2, paw slips versus CoM centrality. Only 5 and 4 mm trials are shown due to fewer slips on 8 and 10 mm ridges. D, effect of ridge width on body alignment. D1: schematic and mean alignment angles of front and hind-body. D2: posture adjustment angle does not correlate with speed. D3, increasing body angles (red, back; green, front) correlate with less central CoM. E, tail-on-body alignment across ridge widths. E1: schematic and mean tail angles with respect to hind-body. E2, larger tail angles correlate with better speed; E3, tail angles do not correlate with CoM centrality. In C, D, and E, values are normalized to ridge width-group means. Data are mean ± SEM; one-way ANOVA followed by BonferroniÀs post-test was used (* $p < 0.01$, ** $p < 0.001$, *** $p < 0.0001$).

Journal of Experimental Biology • Supplementary information

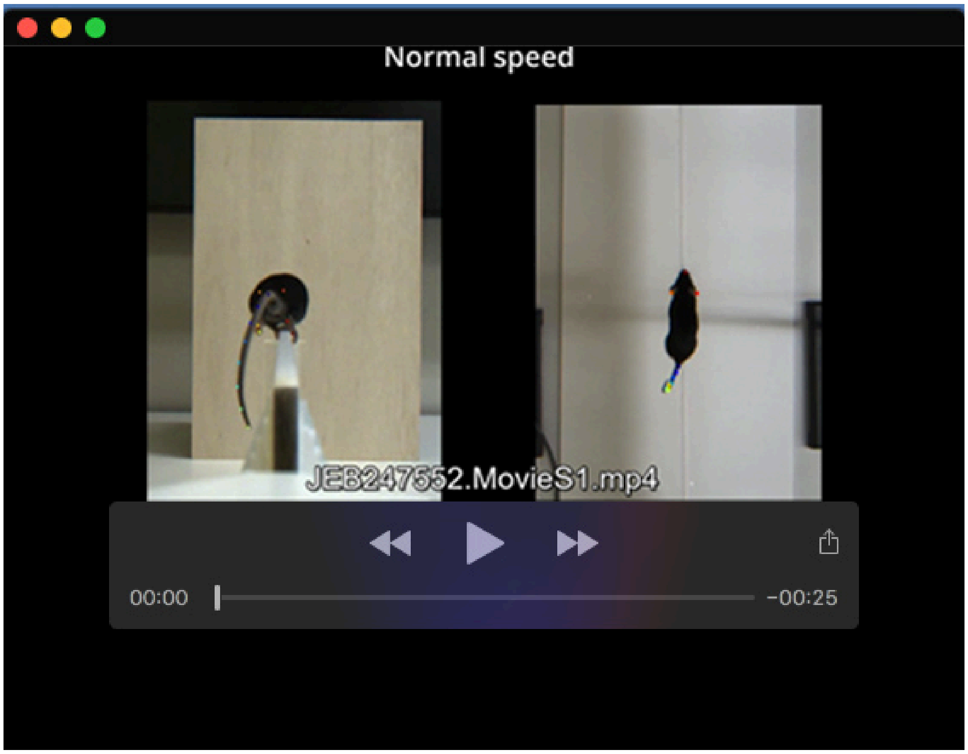

Movie 1. Tail movement evoked by lateral tilting. Normal and 4x slowed-down videos overlaid with DeepLabCut tracking from tilt trials directed contralat erally and ipsilaterally with respect to the tail position.

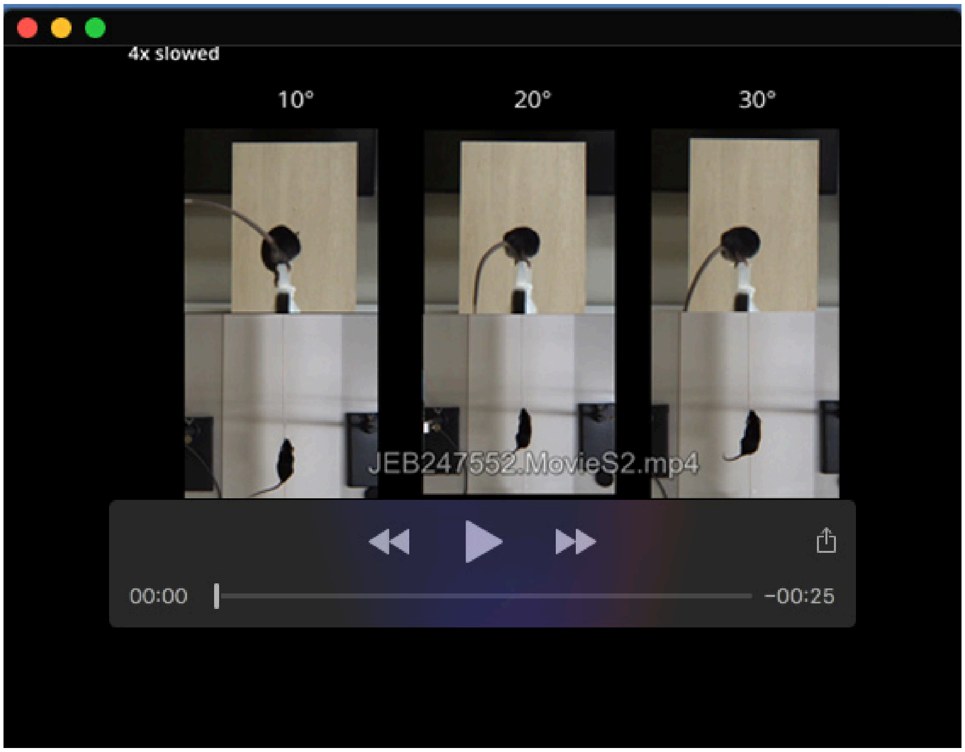

Movie 2. Tail movement evoked by various tilt amplitudes. Ipsilateral per urbation trials with 10, 20 and 30 degree tilts shown in normal and 4x slowed-down video.

Journal of Experimental Biology • Supplementary information

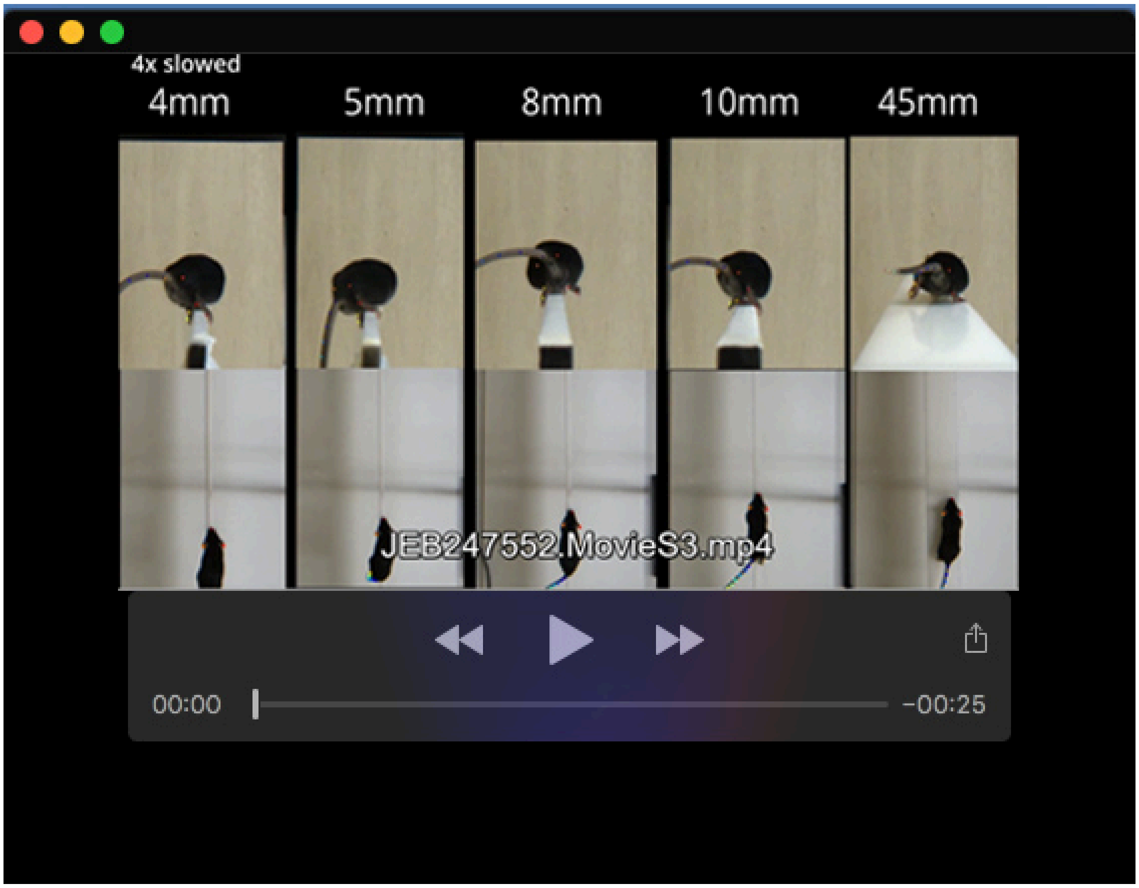

Movie 3. Unperturbed locomotion on ridges of different widths. Same mouse shown side-by-side on different ridges, in normal and 4x slowed-down video.

Journal of Experimental Biology • Supplementary information
